# Learning clinical reasoning in the workplace: a student perspective

**DOI:** 10.1186/s12909-021-03083-y

**Published:** 2022-01-06

**Authors:** Larissa IA Ruczynski, Marjolein HJ van de Pol, Bas JJW Schouwenberg, Roland FJM Laan, Cornelia RMG Fluit

**Affiliations:** 1 Research on Learning and Education, Radboudumc Health Academy, Radboud University Medical Center, Nijmegen, Netherlands; 2 Department of Primary and Community Care, Radboud University Medical Center, Nijmegen, Netherlands; 3 Department of Pharmacology and Toxicology, Department of Internal Medicine, Radboud University Medical Center, Nijmegen, Netherlands; 4 Department of Internal Medicine, Radboud University Medical Center, Nijmegen, Netherlands

**Keywords:** Clinical reasoning, Clinical decision making, Medical education, Workplace learning

## Abstract

**Introduction:**

Clinical reasoning is a core competency for every physician, as well as one of the most complex skills to learn. This study aims to provide insight into the perspective of learners by asking students about their own experiences with learning clinical reasoning throughout the medical Master’s curriculum.

**Methods:**

We adopted a constructivist approach to organise three semi-structured focus groups within the Master’s curriculum at the medical school of the Radboud University Medical Center in Nijmegen (Netherlands) between August and December 2019. Analysis was performed through template analysis.

**Results:**

The study included 18 participants who (1) defined and interpreted clinical reasoning, (2) assessed the teaching methods and (3) discussed how they used their context in order to learn and perform clinical reasoning during their clinical rotations. They referred to a variety of contexts, including the clinical environment and various actors within it (e.g. supervisors, peers and patients).

**Conclusion:**

With regard to the process by which medical students learn clinical reasoning in practice, this study stresses the importance of integrating context into the clinical reasoning process and the manner in which it is learnt. The full incorporation of the benefits of dialogue with the practice of clinical reasoning will require additional attention to educational interventions that empower students to (1) start conversations with their supervisors; (2) increase their engagement in peer and patient learning; (3) recognise bias and copy patterns in their learning process; and (4) embrace and propagate their role as boundary crossers.

**Supplementary Information:**

The online version contains supplementary material available at 10.1186/s12909-021-03083-y.

## Introduction

Clinical reasoning is a core competency for every physician. It is frequently defined as the process where a physician gathers and synthesizes information, generates hypotheses and formulates a clinical impression, prognosis, diagnosis, treatment, care and/or management plan [1]. This is a well-known and time-honoured definition for clinical reasoning. However, this is merely a construct of what we have all once agreed upon in terms of what clinical reasoning is. In clinical practice it is much more complex [1]. Koufidis et al. try to capture this complexity by dividing the concept of clinical reasoning in three conceptualisations [2]. First there is ‘*reasoning as cognitive activity*’, where clinical reasoning can be seen as a sequence of cognitive steps that need to be taken in order to make the right diagnosis. The second is ‘*reasoning as contextually situated activity*’ where clinical reasoning is performed in ‘real world’, unique situations where context needs to be taken into account. The third, is ‘*reasoning as socially mediated activity*’ where learning to reason cannot be seen separately from forming a professional identity and learning to work in communities of practice [2–4].

The learning process involved in clinical reasoning is of considerable interest, given its importance and complexity. Novices start in the pre-clerkship phase, where clinical reasoning is formally trained in medical curricula. During their clinical rotations, students need to apply and further develop all of the knowledge and skills that they have acquired during training, within the workplace environment. Students rapidly notice certain fundamental differences between the practical setting and their experiences in the more theoretical years of the medical curriculum [5, 6]. They experience uncertainty and observe differences between medical departments.

These dissimilarities between theory and practice highlight the importance of clinical workplace-based learning and the numerous patient encounters associated with it in preparing students sufficiently for patient care [7, 8]. Given the rapid change that characterises clinical healthcare settings, supervisors experience increasing time pressure impacting their ability to train and support students. Students who feel less support from their clinical supervisors, therefore, might turn to others (e.g. patients and peers) to improve their clinical reasoning processes [9, 10].

The learning process of clinical reasoning can be seen as a continuum on which a person can grow from a novice to an expert rather than a dichotomous skill where you can either be a novice or an expert. Students find themselves at a different point on the clinical reasoning learning continuum than experts, and as such might be using different learning strategies and processes than experts which we do not fully understand yet [10]. Experts can be far removed from the novice on the continuum, making it more difficult for them to understand the degree of difficulty experienced by the novice in any given clinical problem. This might cause them to give insufficient insight into each step of the clinical reasoning process. In order to gain an understanding of the students clinical reasoning learning processes we investigated the students own understanding of what clinical reasoning constitutes and also their experience of learning clinical reasoning skills throughout the entire medical Master’s curriculum. The results are intended to be used in optimising support for the development of clinical reasoning skills in the workplace. To this end, we formulated the following research questions: (1) What do students understand as clinical reasoning? (2) What helps and hinders their clinical-reasoning learning process in practice?

## Methods

This qualitative study is based on a constructivist approach. We sought insights and deeper meanings in social phenomena through interactions between the participants and the moderator, as well as amongst the participants themselves [11]. We maintained a workplace learning lens whereby learning arises through participation, social interaction and feedback. In doing so, we placed very few restrictions what could be considered to be a part of the clinical reasoning process. We remained open to any existing or new component that could contribute to the clinical reasoning process within the workplace based view [1].

### Setting

This study was conducted at the medical school of the Radboud University Medical Center in Nijmegen, Netherlands. The three-year Master’s curriculum consists of 10 clinical rotations followed in a fixed order by groups of 30 students. During these clinical rotations, students are dispersed across different hospitals. Each rotation is preceded by and concluded with a formal teaching programme, in which the students are reunited as a group. A new group starts the Master’s curriculum each month. Following the launch of a major change in the school’s Bachelor’s curriculum in 2015, an altered Master’s curriculum was introduced in January 2019. For this reason, the school will have two groups of students until 2022: one enrolled in the former Master’s curriculum and one enrolled in the new programme. This situation provided an opportunity to include students from both curricula in this study. The research was conducted between August and December 2019.

### Focus groups

We chose focus groups for this study, as the interaction amongst participants would allow a more in-depth, comprehensive understanding of the topic [12]. Group sizes were kept small enough (4–7 participants) to maximise individual contribution, but large enough to facilitate discussion and generate new insights. To eliminate researcher bias, the interview questions were asked by an experienced, independent moderator from a department other than that of the head researcher (LR), who was nevertheless present as an observer.

### Inclusion

We organised three semi-structured focus groups, each lasting approximately 90 min. The participants were first-year Master’s students who had completed the first year of clinical rotations (Internal Medicine, Neurology, Psychology and Surgery) at the Radboud University Medical Center Nijmegen and its affiliated institutions.

### Data collection

The research group consisted of four medical doctors (LR, BS, MvdP, RL) and one educationalist with a medical background (CF), all of whom had completed their medical training in Nijmegen between 1983 and 2018, with varying clinical experience in various departments (primary care, internal medicine, rheumatology and emergency medicine). All of the researchers are actively engaged in the medical curriculum as teachers (LR, BS, MvdP), curriculum developers (LR, BS, MvdP, CF, RL), clinical supervisors (BS, MvdP), programme directors (MvdP, RL) or as educationalist (CF).

The interview guide was prepared by four researchers (LR, MvdP, BS, CF), based on ‘AMEE Guide No.91: Using focus groups in medical education research’ [12]. The questions were categorised into four major themes: (1) composing a definition of clinical reasoning; (2) attitudes and beliefs on learning and performing clinical reasoning in the workplace and during formal education; (3) the influence of the clinical context on clinical reasoning; and (4) the influence of patients, peers and supervisors on clinical reasoning.

Interviews were audiotaped and transcribed verbatim by a research assistant. The audiotapes were deleted after transcription. The names of the participants were pseudo-anonymised for confidentiality purposes. Only the head researcher (LR) was able to connect data to individual participants.

Ethical approval was granted by the NVMO Ethical Review Board, case number 2018.8.7 (Netherlands).

### Data analysis

We applied template analysis to code the verbatim transcripts from the student interviews [13]. The first template was based on the interview guide and contained five categories: (1) perspectives on clinical reasoning, (2) student characteristics for clinical reasoning, (3) the clinical-reasoning learning process, (4) assessment and (5) ‘other’. This template ensured a focus on themes that needed to be incorporated into the analysis. Four researchers (LR, MvdP, BS, CF) familiarised themselves with the data from one focus group, and then started coding the data, based on a combined inductive and deductive approach [13]. After the codebook was finalised (LR & CF), the transcripts were coded by the head researcher (LR) and one of the other researchers (MvdP, BS, CF). Differences were discussed until consensus was achieved.

In the initial round of analysis, the codes were grouped and analysed by the head researcher (LR) and one of the other researchers (MvdP, BS, CF). Reports of these sessions were discussed (LR, MvdP, BS, CF), reflecting on the outcomes and including additional thoughts from the other researchers. In a second round, all of the outcomes were combined and analysed in a group session (LR, MvdP, BS, CF), which included discussion of data interpretations and placement of the results into the three categories as described by Koufidis et al. [2]. Further modifications to the interpretations were made during the writing process, as well as through continuous discussions (LR, MvdP, BS, CF, RL).

## Results

In total, 18 participants were included in the three focus groups (one with four, and two with seven). A demographic overview is displayed in Table 1. Additional quotations are included in the text (indicated by ‘Q#’) and listed in Table 2.

**Table 1:** Demographical statistics of the participants

Category		N	%
Gender	Male	4	22.2
	Female	14	77.8
Bachelor’s curriculum	< 2015 (old)	6	33.3
	> 2015 (new)	9	50
	Pre-Master’s^a^	3	16.7
Master’s curriculum	< 2019 (old)	11	61.1
	> 2019 (new)	7	38.9

^a^ = Pre-Master’s students are students who have completed a different Bachelor’s degree before they transferred into the medical curriculum. To complete their medical Bachelor’s degree, they enroll in a one-year program consisting of Bachelor’s courses that were not covered by their other degree

**Table 2:** Additional quotations

#	Quote
Q1	*I think that [clinical reasoning] encompasses using your current knowledge in tackling new patients, of new […] uhm […] findings that you encounter. Using that knowledge, you reason what it could be. Because you do not have the experience yet to know what it is, but you do have enough experience to know what it could be and how you could find out.*
Q3	*Yes, I have noticed growth. Especially the past year, during my clinical clerkships. At the beginning I did all the clinical reasoning steps separately. Just like you mentioned, now I’m connecting the steps more. At this point, I’m working on compiling management plans myself and at the next clerkship I want to get more involved in the evaluation, so that I have carried out all aspects of the process. So it’s more about having performed all aspects and doing everything in sequence rather than separately. That’s where I’ve grown. And I’m also doing it more efficiently and in a more targeted manner. At first I found it difficult to see the wood from the trees. But that has become a lot better and I am able to frame things better.*
Q4	*[…]Yeah, then I’ll have the feeling, like ‘I have a responsibility because I’m sitting in a room with this person. I can make this experience a positive or a negative one, purely by how I’m behaving.’ So if you look at it like that, you really have the responsibility, in my opinion. You’re not responsible for the whole medical side of things, but you are for how this patient is feeling at this moment and how he’s experiencing his visit. And I think that […] because of that I’m really motivated to make the most of it. To really immerse myself in their disease, so I won’t miss anything. So that I […] Yeah because you just want what’s best for them or something.*
Q5	*At Internal Medicine, it was like, people were thinking ‘Okay, but what else could it be, let’s think, what’s there? What do we need to know to figure out exactly what it is?’ In Neurology and Psychiatry, and even more so at the Surgery department, it’s more like ‘This is it and we’re going to act on this.’*
Q6	*[…] In Neurology, they would mostly be checking whether it was a neurological problem or not. And if it wasn’t that, it was more or less over immediately.[…] I do find that very difficult when a department says ‘Yes it’s something, we don’t know what exactly, but it’s not something our department should be dealing with, so you have to go somewhere else.[…]*
Q10	*Yes and then you can tell if […] uhm […] ‘Well, was this something I could have thought of myself? Am I lagging behind everyone else?’*
Q11	*And, of course, it’s true that two know more than one, right? So, you might notice that you’re stuck at something because you don’t have enough knowledge of a subject. And then someone who’s sitting next to you knows about different things, so you can get […] then you can work together and still move forward.*

We will divide the results using the three categories as described by Koufidis et al. [2]:

1. Clinical reasoning as cognitive activity.
2. Clinical reasoning as contextually situated activity.
3. Clinical reasoning as socially mediated activity.

### Clinical reasoning as cognitive activity

The students initially appeared to discuss clinical reasoning in a cognitivist manner consistent with their current learning stage as future doctors (Q1). During the course of the conversation, they demonstrated a broader understanding of the clinical reasoning process by including new perspectives. They notice that the process starts before seeing the patient (i.e. by reading referral letters) and that it follows a sequence of steps in a specific order, although it is possible to return to steps to expand information. The clinical reasoning process can end in various types of plans (e.g. further tests, treatment, watchful waiting). And although the basic medical knowledge from their theoretical education forms the basis, it must be supplemented with other aspects, including communication skills, the use of systematic approaches to diagnostic thinking and reliable sources.

*I think that, when you see diagnoses on UpToDate, you’ll ask yourself, ‘Okay, so why does UpToDate point to this diagnosis?’ And then you’ll start to think about it yourself, like, ‘Ah right! So that’s why’. That’s how you train clinical reasoning by using UpToDate. – Q2*.

Students indicated that their development of clinical reasoning strategies consisted largely of performing them more automatically, with clinical reasoning shifting from a process done primarily after the fact to one that became increasingly incorporated into their patient consultations throughout their clinical rotations.

### Clinical reasoning as contextually situated activity

The workplace supports personal growth and growing clinical reasoning expertise. Students mentioned a need to grow into their role as medical interns before being able to grasp their own learning processes in the workplace. By observing people while working in clinical practice, they learned how to handle complex clinical situations (e.g. coping with the fact that even trained physicians do not always have all the answers, and there is not any single correct way to treat patients). Asking critical questions was mentioned by students most as a tactic used to start a discussion about the clinical reasoning process with their supervisors. By asking questions, they were able to test their own thoughts compared to those of their supervisors and consequently either confirming or broadening their own perspectives.

Students expressed a need for support and structure that support their participation in practice, such as knowing what they can and must do in practice, what they can get out of the workplace as learners, and what is expected of them as interns. They reported the necessity of: daring to ask questions, making mistakes, taking control, being confident, speak up, make choices and indicate boundaries and/or uncertainties.

The mentioned aspects of clinical reasoning that the students learned at the workplace were (1) encountering cognitive bias (e.g. tunnel vision and thinking in silos); (2) developing clinical intuition (e.g. ‘a gut feeling’); (3) strengthening clinical reasoning skills (e.g. building up illness scripts, working more efficiently); and (4) taking increasing responsibility for the clinical reasoning process.(Q3) The students defined a number of facilitators required for excelling at clinical reasoning during their clinical rotations: a solid base of basic knowledge (of physiology, pathophysiology, anatomy and epidemiology), knowing where to find and use reliable sources (both online and offline), knowing how to interpret physical examination, knowing one’s own strengths and weaknesses and learning from different patients.

To prevent errors or cognitive bias, students used the technique of constant comparison and evaluation. Other techniques included the ‘review of systems’ when taking histories in order to prevent tunnel vision, discussions with patients or others, and drawing on their own flexibility when confronting changing or new situations. The students identified formal learning activities and clinical workplace activities as valuable educational tools for clinical practice, provided that they met certain conditions (Tables 3 and 4).

**Table 3:** Conditions for valuable formal learning activities

*Students identified ****formal learning activities*** *as valuable and educational for learning clinical reasoning if...*
…they are interactive, especially when the main goal is to start a discussion
…they allow explicit practice of the steps of clinical reasoning
…they allow cooperation with peers, either in small or in large groups
…they allow for observation of others (peers, teachers) when performing clinical reasoning
…they simulate clinical practice as realistically as possible (e.g. through the use of simulated [or real] patients or patient descriptions with extensive patient context)
…they allow practicing skills in new situations

**Table 4:** Conditions for valuable workplace learning activities

*Students identified learning activities as helpful for learning clinical reasoning ****in the workplace*** *if*...
…they offer the opportunity to perform clinical reasoning independently, either with or without supervision
…they allow the direct application of knowledge in clinical practice
…they involve observation by and interaction with a supervisor
…they involve students being ‘thrown in at the deep end’ and forced to step outside of their comfort zone
…they include receiving feedback
…they involve the repetition of known diseases and gaining experience in as yet unknown diagnoses

Students noted that their clinical rotations helped them to integrate and connect theoretical knowledge with clinical practice. They observed that the workplace had a positive influence on their motivation to perform clinical reasoning. They further expressed a desire to invest additional effort to deliver qualitatively good care to patients in clinical practice (Q4).

During their rotations, the students discovered remarkable differences between styles of clinical reasoning across the various medical specialties. For example, in the internal medicine department, the clinical reasoning process was relatively explicit, while it seemed to go much faster and more implicitly in the surgery department (Q5). In the psychiatry department, history-taking and psychiatric examination are intertwined, which influences the clinical reasoning process. In contrast, the boundaries of the specialised domain were quite strict in the neurology department (Q6).

### Clinical reasoning as socially mediated activity

Students reported that social interactions within the workplace setting with different actors was important for the development of their clinical reasoning skills.

#### Supervisors

Students described how supervisors contributed to learning clinical reasoning in clinical practice in two major ways: (1) making the clinical reasoning process visible and (2) creating a learning environment (whether safe or unsafe). According to the students, learning occurs predominantly through role modelling and feedback.

*I like it when supervisors make their own process explicit. Because you have supervisors who […] who make learning very dynamic. You also have supervisors […] who don’t do anything at all actually; they find it annoying when they have to supervise a student, and they want it to be over and done with as quickly as possible. And you have supervisors who […] uhm […] sometimes they don’t know what to do exactly, but they’ll just explain what they are thinking the whole time. Even that is very educational for me. So I find that a very nice method as well. So either ‘challenge me, ask questions and make me think’ or just show me ‘this is what I do’. – Q7*.

#### Patients

The students indicated that patients had influenced their clinical reasoning in several ways. First, they contributed to the clinical reasoning process, especially the diagnostic process, by providing students with important information concerning their symptoms, as well as by providing explicit or implicit feedback on how the process was going so far. Students further mentioned that they worked together with patients on various steps in the process.

*But sometimes you’re able to work together with a patient for a bit […] that you really […] that you’re cooperating in the search together […] puzzling together. Some patients actually enjoy doing that. – Q8*.

Secondly, students reported that patients had influenced their cognitive processes as well. For example, they noted that the constantly varying contexts of individual patients could trigger tunnel vision or other forms of bias. Individual, patient-specific contexts enhanced the learning process by requiring customised modifications in history-taking, reasoning and compiling a management plan. This category also included character traits and patient-specific behaviour.

*“Well, if you are thinking: ‘I have found something and this is the best choice. or let’s say. I want to give the patient these pills.’ And the patient tells you: ‘I really, absolutely hate pills and I won’t take them’. Then you’re still nowhere, while there may be some other treatment options that you can turn to.” – Q9*.

Finally, they mentioned that, in contrast to written patient cases, real patients brought out the best in them, requiring them to think outside of their own medical specialisations to help steer patients in the right direction (Q4).

#### Peers

Students compared their own clinical reasoning to that of their peers in order to evaluate their own performance (Q10). They also talked with their peers about learning and clinical reasoning during educational activities, as well as in the workplace. Reviewing the process together or simply telling each other about their day-to-day experiences and educational moments are two of the activities that students identified as beneficial to their own learning. Of equal importance was the pleasure of learning and working together. Students also learned from the exceptional situations that their peers encountered and recounted, as they stored such knowledge and referred to it when encountering similar situations. Finally, they mentioned turning to each other for help when they became blocked in their own process (Q11).

*Sometimes you were sitting there by yourself, and sometimes you were with four other people. And then you’ll talk about what you’ve seen that day. And you’ll share your experiences and automatically also your learning process. And then you can learn from someone […] well not only from someone’s mistakes but also just from what someone has done right. – Q12*.

One aspect that was identified as counterproductive was the perception of a competitive atmosphere within the peer group, which discouraged students from asking questions, asking for help and sharing information.

## Discussion

This study provides insight into how students perceive clinical reasoning and how they develop their own clinical reasoning skills in daily practice during clinical rotations. Students improve their clinical reasoning skills by working together with supervisors and each other, as well as by seeing a variety of patients with similar and differing diagnoses. Considering previous research on workplace learning [10, 14], our results provide new insight into the relevance of the work/learning context and specific elements of that context for clinical reasoning.

We found that students frequently use each other as sparring partners by comparing and contrasting their own performance. They recognise that clinical reasoning can be a group process as well as an individual process. The effect of peer-assisted learning during clinical rotations has been extensively studied by authors including Raat et al. and Tai et al. To date, however, scholars have not provided any explicit description or investigation of the specific gains in clinical reasoning in this regard [8–10, 15–17]. This research indicates that there is still more to explore about the ways in which students use peer-assisted learning for developing their own clinical reasoning skills at the workplace. Our findings indicate that interactions with patients can contribute to the improvement of clinical reasoning skills. It is known that patients play a more implicit (passive) role in students learning processes [10, 17], but according to our results, their role could be expanded to an explicit (active) role as well since our students assign priority to patient-centered care by partnering and ‘puzzling’ with patients in the process.

Our results agrees with existing research in finding that students perceive differences between clinical reasoning as presented during their pre-clinical education and as experienced in their clinical rotations. Koufidis et al. describe these differences as ‘disjunctions’: ‘tensions experienced by the learners in a clinical situation when perceived elements in the situation appeared inconsistent with previous experience, unexpected, self-contradictory or unfamiliar’ [6]. Our research adds a new disjunction to their list: the difference between departmental boundaries as experienced by students in clinical practice, as compared to the manner in which they are educated about these boundaries during their formal education. These differences could arise given that pre-clinical education tends to focus on patient-centred care, holistic medicine and interdisciplinary work, while the working environments to which students are exposed during their clinical rotations tend to be of a more mono-disciplinary character. This could make students aware of differences in the ways in which clinical reasoning is performed across medical specialisations, often making them aware of difficulties relating to boundaries that are often quite distinct. The organisation of clinical rotations allows students to become members of specific communities of practice for short periods [18, 19]. Each community of practice entails both domain-specific clinical reasoning skills (e.g. illness scripts and problem-solving procedures) and generic skills (e.g. organization of knowledge). Close to the boundaries, in the peripheral areas of a specific community of practice, students find fertile ground for training both domain-specific clinical reasoning skills and generic skills [19, 20]. When moving from one department to another during clinical rotations, students frequently cross the monodisciplinary boundaries. We believe that both students and supervisors could benefit more from such boundary-crossing activity. Supervisors could use students in their own clinical reasoning process, for example, when seeing patients whose symptoms cross the boundaries of their discipline and who need a more multidisciplinary approach. In turn, students could distribute useful knowledge across the landscape by engaging in dialogue with their supervisors. Given the brevity of their community membership, combined with the written or unwritten rules of specific communities, however, students may often feel uncomfortable taking on the roles of boundary crossers and knowledge propagators within these ‘landscapes of practice’.

In this context, students often appear to identify biases (e.g. tunnel vision and thinking in silos) in their supervisors. As experts, supervisors tend to skip steps in problem-solving, which they fail to make explicit to their students, who are still novices [21]. This discrepancy between novices and experts—which can be explained through the knowledge restructuring and encapsulation that takes place as students develop into clinical experts by passing through different domains—should be made explicit [20]. Another important role that supervisors can use to improve the clinical-reasoning learning practice involves facilitating a learning environment that allows students to participate in the valuable, educational and helpful learning activities mentioned in Tables 3 and 4.

Our results are remarkably consistent with those of previous studies that have recommended the implementation of dialogue in the teaching and learning of clinical reasoning [10]. One reason that ‘using dialogue’ has yet to be fully integrated into daily practice could be that the notion is too vague. We therefore present several practical implications of our results, in order to improve the clinical-reasoning learning practice through dialogue at three levels.

First, at the individual level, students could benefit from greater empowerment to start conversations with others (e.g. peers, supervisors), which could help them to make better use of their role as boundary crossers, to clarify their observations in the workplace and to increase their inclusion of patients in their clinical reasoning process. Second, at the instructional level, supervisors should think beyond the boundaries of their own disciplines and use dialogue with their students as a method to help them make their own problem-solving process explicit. Finally, at the group level, conversations amongst students should be encouraged in order to promote peer learning and create a deeper understanding of the clinical reasoning process.

With these recommendations, our study builds upon existing learning theories and introduces new ideas for future research or interventions aimed at improving the clinical-reasoning learning practice of medical students. The understanding of how this learning practice actually works can be increased by allowing students to have a voice in the process.

### Strengths and limitations

One limitation of this study is that the focus groups involved students from only one institute, based on a cross-sectional selection at one point in the curriculum (first year of the Master’s programme, after the first four rotations). Interviewing students from other institutes might have provided a broader view. Because our students had completed their clinical rotations at several different hospitals, which provided different levels of secondary or tertiary care, each with its own teaching programme, we were nevertheless able to obtain input from a variety of teaching hospitals. To broaden our perspective, we drew on internationally recognised and applicable educational theories for analysing the data, and we included students from both the former (more traditional) and the new curriculum.

In all, we obtained data from 18 participants across three focus groups. Although this is arguably a small number, the interviews generated a large volume of information. We therefore concluded that the data set was rich enough to ensure sufficient conceptual depth for answering the research questions [22].

One strength of this study concerns the composition of the research team. The first researcher is a young doctor who recently completed graduate studies, such that the student perspective was still quite familiar. In addition, the involvement of an independent moderator for the interviews helped to create a safe environment in which the students felt they could speak freely. Other members of the team have backgrounds in both medicine and education. Finally, the involvement of the entire team in the discussions concerning data interpretation ensured the inclusion of a variety of perspectives throughout the research process.

## Conclusions

With regard to the clinical-reasoning learning process of medical students in practice, this study stresses the importance of integrating the practical context into the clinical reasoning process and the way in which it is learnt. The full incorporation of the benefits of dialogue into clinical reasoning practice will require greater attention to educational interventions that empower students to (1) start conversations with their supervisors, (2) increase their engagement in peer and patient learning, (3) recognise bias and copy patterns in their learning process and (4) embrace and propagate their role as boundary crossers.

## Supplementary Information

**Additional file 1.**

## Data Availability

The datasets used and/or analysed during the current study are available from the corresponding author on reasonable request. Standards of reporting: All methods were performed in accordance with the relevant guidelines and regulations.
