# Supplementary material for: Learning clinical reasoning in the workplace: a student perspective
Source: BMC Med Educ. 2022 Jan 6;22:19. doi: 10.1186/s12909-021-03083-y (PMC8740056; doi:10.1186/s12909-021-03083-y)
Supplement: Supplementary file 1 — Additional file 1. [file 12909_2021_3083_MOESM1_ESM.docx]

**Interview Guide**

***Opening questions*** *(ask for facts, not attitudes or opinions)*
- Where have you done your clinical rotations so far?
- Did you do so alone or with others? If with others, were these the same people?

***Introductory questions*** *(open-ended questions to introduce the topic)*
- What is your understanding of clinical reasoning?
- Did you receive any education on clinical reasoning? What kind of education? When? (theory/practice)
- How are your clinical reasoning skills formally and/or informally assessed? (theory/practice)

***Transition questions*** *(same line as introductory questions but delving more deeply into the topic)*
- Could you give a practical example of someone performing clinical reasoning **well**? (When? What did this person do? How? Where? What sort of impact did this have on you?)
- Could you give a practical example of someone performing clinical reasoning **not so well**? (When? What did this person do? How? Where? What sort of impact did this have on you?)

***Key questions***
- How did you learn clinical reasoning? In what way do you apply in practice what you have learned in class? What do you think would be the ideal way of acquiring it?
- In what way does practice offer you opportunities for acquiring clinical reasoning skills? (what is the role played by practice in learning?)
- What knowledge, attitudes and skills should someone possess to be able to perform clinical reasoning well? Do you have any role models? What would your role model look like?
- Have you gone through a development in your clinical reasoning skills? How can you tell? Were there any points when you made a leap forward? What have you done to improve your skills? What has been the influence of your supervisors? What has been the influence of your peers? What is the influence of context? What is the influence of education? What is the influence of patients?
- What would be the best way for a supervisor to support you in improving your clinical reasoning skills in practice? What is unhelpful in supervision?
- How would you like to be assessed on your clinical reasoning skills?

***Concluding questions*** *(summary and conclusion)*- Thanks very much for your time. Is there anything that we have not discussed and that you would like to say?
